# The Fifth Session of the Research Society of the American Red Cross in France

**Published:** 1918-05

**Authors:** 


### The Medical Bulletin

N° 7 PARIS MAY 1918

### RESEARCH SOCIETY REPORTS

### THE FIFTH SESSION OF THE RESEARCH SOCIETY OF THE AMERICAN RED CROSS IN FRANCE

April 19 and 20, 1918, at the Hotel Continental, Paris.

Major WALTER B. CANNON, Chairman of the Research Committee, presided throughout the session.

The subject of the first meeting, Friday, April 19, at 2:00 P.M., was Dysentery (Bacillary and Amebic).

Lieutenant-Colonel CHARLES J. MARTIN, Consulting Pathologist, A. I. F., read a paper on the " Diagnosis of Bacillary Dysentery ".

Colonel Martin dwelt on the limited usefulness of bacteriological diagnosis in bacillary dysentery. He said that at the outset of the war bacteriological methods were relied upon in the Austrian and German, as well as in the British armies. Dorendorf and Kolle, dealing with an epidemic on the Russo-German front, isolated B. dysenteriae Shiga from so few specimens that they concluded, notwithstanding the general clinical resemblance of the epidemic to dysentery of this type, that the outbreak was due to some other cause. Other German writers seemed to corroborate the fact which the Australians had discovered in the Dardanelles in 1915 and confirmed in Egypt and Palestine in 1916, i. e., that the chance of recovering one or other of the dysentery bacilli was small unless the dejecta came under examination in the first few days of the disease.

The author, in conjunction with F. E. Williams, A. A. N. S., from June to December 1917 made upwards of 1200 attempts to recover dysentery bacilli from the stools of patients at various periods of their illness. Results showed that the chance of isolating dysentery bacilli rapidly diminished after the first few days. This appeared to be the case whether the stools remained characteristically dysenteric or not. The method employed was to plate out a suitable dilution of the stool, if homogeneous, upon a plate of MacConkey's lactose-bile-salt-peptone-agar. If possible a piece containing mucus was chosen, washed and broken up in sterile water, and some drops well rubbed over on the plate with a glass rod. If the piece selected did not produce about 500 colonies a second plate was made next day. From 18 to 20 hours were allowed for growth, and samples of the dysentery colonies (if results were positive) were then sown into tubes of warm broth. Four hours later they were examined for motility and sown into tubes of peptone water containing 1 o/o of lactose, glucose, and mannite respectively, and also on the surface of an agar slope. If, after incubation all night, the actions in the various carbohydrate media were typical, the growth on the agar slope was emulsified and its agglutinability tested against dilutions of appropriate agglutinating sera. It was rare to find B. dysenteriae the preponderating organism on the plates, though this occurred sometimes when perfectly fresh muco-pus obtained from a patient in the first or second day of the disease was the material sown. Usually, when dysentery bacilli were present, the number of colonies varied between 1 and 20 on a plate showing 500-1000 other organisms.

Experiments undertaken to ascertain whether MacConkey's medium offered any hindrance to the growth of B. dysenteriae Shiga or B. dysenteriae Flexner, showed that the bile salts contained in it did not prevent the colonies of dysentery bacilli from appearing though they were of smaller size.

Abbreviation of the technique does not seem to be permissible, and resort to agglutination tests without testing for the essential cultural and biochemical characteristics of one or other of the dysentery bacilli is dangerous, particularly in the case of the mannite fermenting group of dysentery organisms, for high titer sera prepared against this group are not specific in moderate dilution and will agglutinate several kinds of coliform bacteria and cocci. Ignorance of this fact has led to much confusion in some military laboratories, both in our own and enemy armies. The muco-pus and epithelial débris discharged in the milder pathological condition, and the necrosed coagulated superficial layers of the mucous-membrane in the more serious, are, except in the earliest stage of the disease, invaded by hosts of intestinal organisms which soon overwhelm the dysentery bacilli originally present. This places a serious limit to the value of a direct bacteriological diagnosis of dysentery.

Colonel Martin then discussed the value of agglutination in the diagnosis of dysentery. He had, in collaboration with Captain

Hartley, R. A. M. C., and F. E. Williams, A. A. N. S., examined the sera, between the tenth and thirtieth days from the onset, of a number of cases of dysentery from which the B. dysenteriae Shiga and the mannite-fermenting group of bacilli (13 and 151 respectively) had been recovered, and also of a number of control persons (214) who had no history of dysentery.

The method employed was that recommended by Major Dreyer, viz., keeping equal volumes of the emulsions and progressive dilutions of the sera in a bath at 55° C. for 4 1/2 hours, and reading after 15 to 30 minutes. The emulsions employed were supplied from Prof. Dreyer's laboratory at Oxford. A degree of agglutination which could be comfortably seen with the naked eye against a black background, corresponding to Dreyer's "standard agglutination," was taken as the end point. The results confirmed those of Dreyer, and indicated that the presence of 10 or more agglutinin units per cubic centimeter of serum is for all practical purposes diagnostic of an active bacillary dysenteric infection caused by the bacillus agglutinated, and that the presence of 7 units is highly suggestive of dysenteric infection. The error introduced on account of the reaction of the serum of normal persons to the standard agglutinable cultures used amounts to 2 1/2 o/o in the case of B. dysenteriae Flexner and to 2 o/o in the case of B. dysenteriae Shiga infections.

The author's group of cases due to infection by B. dysenteriae Shiga was a small one, but in every case of proved Shiga the patient's serum agglutinated the standard agglutinable culture used, and this agglutination was always complete in the highest dilution of the patient's serum tested. The results seemed to indicate that in the case of infections due to B. dysenteriae Shiga determination of the agglutinin content of the patient's serum constitutes a valuable method of diagnosis. This is, however, far from true with patients infected by members of the mannite-fermenting group of dysentery bacilli. Less than 40 o/o of persons who had recently been infected by one of the Flexner Y. groups exhibited an agglutinin content in the serum of 10 or more units. The low proportion observed might have been due to the fact that in many patients the infection was very mild and the agglutinins produced so small in amount that they were not demonstrable; but in the light of the well ascertained fact that there is a family of more or less discrete strains of mannite-fermenting dysentery bacilli, it appeared more likely that some patients had been infected by one member of the group and others by another.

To test this, emulsions were prepared of four other strains of Flexner bacillus, 0.1 o/o formalin was added, and they were kept in the ice box for a week prior to use. The agglutinating power of the sera of 40 proved cases of dysentery and of 40 control persons was determined against each of these as well as against the "standard agglutinable culture," using Dreyer's technique. Of the positive sera tested :

11 failed to agglutinate any of the 5 emulsions used;
29 agglutinated one or more of the 5 " "
2 " all 5 emulsions;
4 " 4 " only;
3 " 3 " "
7 " 2 " "
13 " 1 " "

Results showed that if the sera of patients who had recently suffered from infection by a member of the Flexner group of dysentery organisms were tested against five strains instead of one, the percentage giving a positive result increased from 40 to 72.5; and it is reasonable to suppose that the number yielding a positive result would be still further increased if the patients' sera were tested against a still greater number of locally isolated strains. Such increased complexity, however, would rob the method of its practical value.

Bacteriological methods are unsatisfactory for the routine diagnosis of bacillary dysentery. When, notwithstanding the labor involved and the resources of the well-equipped laboratory, a result is arrived at which compares unfavorably with that obtained by the observation of the patient and of his evacuations, one wonders whether a simplification of procedure would not be more efficient. The author is of the opinion that if clinical observations be supplemented by the microscopical examination of dejectae (for some innocent-looking stools contain much pus), dysentery can be diagnosed in 95 o/o of instances. Microscopical examination of stools passed at different times will also determine approximately which cases are amebic in origin. The remainder may, with a small margin of error, be regarded as bacillary. Neither for the disposal of the patient nor for his proper treatment is it material whether he be suffering from an infection by B. dysenteriae Shiga or by one of the mannite-fermenting group of dysentery bacilli. Should it be desirable to ascertain this in connection with an outbreak it can be done by the bacteriological analysis of a few suitable examples from early cases.

Major F. Rist, of the French Reserve Medical Corps, read a paper entitled " Bacillary Dysentery in France During the War ". He told of his experience in August 1916 with an outbreak of bacillary dysentery among the employés of a large powder factory. Several cases of acute diarrhea with bloody mucous stools were reported. The conditions were ideal for an epidemic, for there was a large mixed population of soldiers, civilians, (men and women) and foreigners (Serbian, Italian, and Indo-Chinese), some of whom lived in huts and others boarded with the neighboring villagers who supplied Tours and the vicinity with dairy and farm produce. The factory had outgrown its sanitary arrangements, and the workers did not confine themselves to the use of the proper facilities. The neighborhood was infested with flies.

Probably the disease was carried from the front into the interior by soldiers on furlough. These men had recently occupied German trenches, where they had in all likelihood been infected, for during the year 1916 there was an epidemic of bacillary dysentery in the German army and in the civil population as well. The death rate in Prussia among those infected was 10 o/o. Probably it was slightly higher in the army. It is certain that the disease was carried into the Brittany district by German prisoners taken on the Somme.

During the epidemic in the Touraine district the death-rate in the military hospitals among those infected was 2.8 o/o; and among the civilian population, 11.4 o/o of those who had dysentery died.

The dysentery bacillus was isolated and identified by the ordinary method of plating some of the previously washed mucous matter from the fresh stools in lactose litmus agar. Non-motile, gram-negative, non-fermenting bacillary colonies were then tried in other sugars, mannite, and maltose, and agglutination tests were made with powerful, agglutinating, immune sera prepared at the Pasteur Institute against the Shiga and the Flexner types of dysentery bacillus. It is useless to try to isolate the dysentery bacillus unless the stools are fresh.

One of the baffling conditions in the epidemic arose from the fact that light cases are the most numerous, and such cases rarely come to the doctor's attention.

The typical Shiga bacillus was isolated in 61 o/o of the cases observed. In 22 o/o of the cases, however, a bacillus was isolated which had all the cultural and fermentative properties of the Shiga type, but which was not agglutinable by a Shiga serum. In the remaining cases the Flexner and Hiss types were isolated. It has been observed that in an epidemic of dysentery the same organism is rarely to be found in all the cases. In the majority of the severe or fatal cases the Shiga type was the cause, but there were also fatal cases in which the Shiga type did not occur. The speaker stated that he was convinced that the agglutinative test had very little practical diagnostic value, and that it was often misleading.

The antidysenteric serum prepared at the Pasteur Institute had proved very satisfactory during this epidemic. It must be given in large doses subcutaneously : 40 cc. daily in moderately severe cases, and 80 or 100 cc. for very severe cases. Much harm is occasioned by physicians who insist upon giving the routine treatment of diarrhea. During an epidemic serum-therapy should be the common practice.

High doses of anti-dysenteric serum seemed wonderfully effective, curing cases in a very short time.

Among the interesting features of the epidemic of 1916 the speaker mentioned the great frequency of rheumatoid swelling of the joints, which became exceedingly painful. The swelling was often very pronounced. This was a tenacious and troublesome complication. Dysenteric rheumatism was never coincident with the dysenteric stage of the disease. Rheumatism might not appear until the day when the stools again appeared normal, or even two weeks later.

In some of the cases, the dysenteric stage completely escaped the physician, and only the rheumatism was observed, so that it sometimes appeared as if there were an epidemic of rheumatism.

Post-dysenteric conjunctivitis was also very conspicuous. It was characterized by injection and redness of the bulbar conjunctiva, with very little secretion and very moderate pain. It was almost always associated with dysenteric rheumatism.

Major A. W. M. Ellis, N° 5 (Canadian) Mobile Laboratory, pointed out the importance of bacillary dysentery as a war disease and mentioned the important part it had played in other wars, and in the present campaign.

Bacillary dysentery first appeared extensively in the British army in France during the fighting in the Somme area in the summer of 1916, since which time it had been constantly present. During 1917 the number of cases had been smaller than in 1916 and had occurred chiefly in two epidemic areas, one a large infantry base dépôt and the other in the Second Army. The experience of the speaker was confined entirely to the last-mentioned epidemic in the Second Army.

This epidemic started in the third week of July among the men at the wagon-lines of a battery of Field Artillery. During April, May, and June no cases of bacillary dysentery had been reported from this area and on the occurrence of the first case the speaker had at once visited the unit to investigate the probable source of infection. At this visit thirteen men suffering from diarrhea, but not considered sufficiently ill to warrant sending to hospital, were discovered. These thirteen men were at once evacuated to hospital and their faeces examined bacteriologically. From 11 of the 13 B. dysenteriae Flexner-Y was isolated. During the following week eight further bacteriologically positive cases occurred in this unit, and within ten days a diffuse outbreak, affecting almost every unit, occurred over the whole corps area. In spite of the most strenuous endeavor this area has not since that time been freed from bacillary dysentery.

The arrangements for controlling the epidemic consisted in efforts to improve general sanitary conditions and arrangements for the prompt evacuation and investigation of all cases of diarrhea. For this purpose a segregation camp of 250 beds was established some five miles behind the line in close touch with the laboratory, and instructions were issued that all cases of diarrhea of 24 hours' duration should be evacuated to this camp. From August 1917 to April 1918 all cases of diarrhea sent to Field Ambulances in the southern half of the army area thus passed through one unit under the clinical observation of one or at most two men, this clinical observation being in every case controlled by bacteriological examinations. Examinations of two specimens of faeces were carried out in every case.

Of 5332 cases of diarrhea examined bacteriologically by the speaker and his assistant, Capt. Wm. Warwick, C.A.M.C., 683 or 13 o/o were bacteriologically positive, while 907 or 17 o/o were clinically dysentery though negative bacteriologically. 1589 or 30 o/o of the 5332 cases of diarrhea were therefore evacuated to the base as dysentery. Of the 683 cases from which dysentery bacilli were isolated, only 10 were due to B. dysenteriae Shiga, the remaining 673 being all due to infections with the Flexner-Y group, the proportion being roughly Flexner: Y : : 2 : 1. The only other definitely proved pathogens isolated from these cases of diarrhea were Para B (seven instances) and B. typhosus (one instance, history of typhoid of years previously).

The speaker went on to consider the epidemiology of dysentery and pointed out that the common practice of considering bacillary dysentery and enteric group infections as comparable epidemiologically was possibly entirely wrong. In enteric group infections the patients were almost always so ill that they could not possibly carry on. All cases were therefore automatically rapidly removed from their units and the source of infection thus eliminated. In enteric group infections the chronic carrier was therefore, as regards the army at least, the important and dangerous source of infection. The speaker believed that, in bacillary dysentery, conditions were entirely different, in that with this disease many very mild cases occurred — cases so mild that frequently, perhaps even usually, they were never evacuated from their units. In bacillary dysentery these mild cases, the speaker believed, were the common means of spreading and continuing epidemics, and he felt that if the great amount of work and effort which had been spent in detecting and controlling carriers had been applied to the detection and control of these mild cases more good would have been accomplished. The usual history of an outbreak of dysentery in a unit was as follows. A unit having no diarrhea, or perhaps one or two cases a week, would suddenly one morning have five, ten, or even fifteen men on sick parade with diarrhea. Of these one or two would perhaps be sufficiently ill to require evacuation to Field Ambulance. The remainder would be given a purgative, would appear on sick parade again the next morning, receive a sedative and carry on without further serious discomfort. From then on, however, you would find the unit never free from diarrhea. Every morning there would be a fresh case, perhaps two or even three, until finally more severe cases would occur and would prove to be bacillary dysentery. The speaker had seen this sequence repeatedly and had seen the diarrhea and dysentery disappear from the unit as soon as the recognition of the fact that the unit had dysentery led to thorough evacuation of all cases of diarrhea. This was the difficulty : the mildness of many of the cases and the difficulty in persuading medical officers and commanding officers to evacuate men who obviously were not seriously ill. The speaker's experience had been gained in an area in which everybody was doing their utmost to help and cooperate. It was, however, impossible to get cases evacuated without showing the reason why, and so long as they were only " diarrhea " the reason could not be proved. If some scheme could be devised to prove that these mild cases were dysentery, a definite step in the forward direction would be made.

A paper by Doctor Paul Ravaut, Médecin-Major de 1re Classe, entitled " Amebic Infection in France During the War ", was read in translation. The writer stated that in 1915 amebic infection presented itself as a perplexing problem hitherto unknown in France. Even men who had never been outside of France showed various bacilli in their stools. In spite of strong doses of anti-dysenteric types of dysenteric serum, the effects on the patients were practically nil. After a systematic examination of the stools, however, the presence of amoebae showing all the characteristics of Entamoeba dysenteriae was detected. The emetine treatment gave excellent results.

In the diagnosis of dysentery, both parasites should be borne in mind, and the predominance of one or the other demonstrated. The occurrence of a large number of cases of autochtonous amebic infection, contracted simultaneously in a relatively limited area, shows that this disease may not only thrive in our climate, but may even assume epidemic proportions.

In 44 o/o of the intestinal cases treated in the north we found amoebae or cysts in the stools.

The origins of the contagion are many and are easy to determine. It arises in certain African and colonial regiments. French troops were contaminated at the Dardanelles, in Syria, in Macedonia, and on the French front, by being brought into contact with the afore-mentioned troops. As the disease is easily contagious in our climate, it has spread with great facility. Amebic cysts have great powers of resistance and remain for a long time in a latent state. They are found not only in old sufferers from the disease, but also in healthy subjects.

Amebic dysentery behaves like many other infectious diseases common in our climate and the rôle of carriers becomes as important in the propagation of this disease as in most of the others. In its epidemic form, it may be regarded as a new disease in France.

Amebic dysentery assumes the form of an essentially chronic disease, marked from time to time by acute phases constituting the attacks of dysentery. Very acute forms, however, have been observed, running a course similar to that of an infectious illness.

Chronic amebic dysentery may sometimes follow an acute attack of typical dysentery, but in France these initial stages are more often mild.

To these symptoms must be added signs of physical deterioration, of suprarenal inactivity, or of inflammation of the liver. The older and the more fatigued the patient, the more rapid are the effects of the disease, which is sometimes complicated with hepatic symptoms.

Extensive abcesses of the liver have been relatively rare. On the other hand simple congestion of the liver has very often been observed. Between these two forms, intermediate forms are frequently demonstrated.

All the hepatic cases had previously suffered from intestinal troubles, though never of clearly dysenteric character.

The clinical symptoms of hepatic involvement are chiefly a slight arching of the thoracic cage, a definite well-marked pain accompanied by subcutaneous edema, a limited increase in the volume of the liver, and almost always, a moderate amount of fever.

Radioscopic examinations are particularly important for the control of limited hepatitis with swelling of the upper surface of the liver. Examination of the pus appears also to be very important. So long as the pus is bright red and contains well preserved leucocytes, it is possible to obtain reabsorption of the abcess by medical treatment.

During the course of the hepatitis the search for amoebae or cysts in the stools is deceptive and even contradictory. The condition of all such cases is considerably improved by medical treatment. Hepatic deformation, fever, and pain, rapidly disappeared under the influence of the emetine and arsenic combination.

The size of the cyst alone does not constitute a reliable line of demarcation between the two varieties and the maximum adopted was 14.5 $\mu$ for those containing the B. dysenteriae and 17 $\mu$ as the minimum for those containing the B. coli.

When examining uncertain forms of amoebae it is sometimes helpful to produce movements, as can be done by rapidly heating the slide with a match.

As far as diagnosis is concerned, two hypotheses are possible: either there is an association of amoebae and bacilli (in which case the bacilli are of secondary importance) or else there is a "camouflage" of the amebic dysentery by the bacillary. The correctness of the interpretation is proved therapeutically.

Amebic dysentery was not recognized because it was not expected and because, until the war, it was too often considered to be a disease peculiar to hot climates. The famous phrase of P. Manson, that "the great secret of a successful diagnosis of hepatic abscess is to suspect it", was never more completely verified.

Another reason for the failure to recognize amebic dysentery was that very often dysenteric symptoms were absent and neither amoebae nor cysts could be demonstrated in the stools. We know that their elimination, particularly in the case of cysts, is intermittent. If none of the usual methods of diagnosis give positive results, nevertheless amebic dysentery should be suspected.

The amebic infection is chronic, subject to sudden acute attacks, and is due to protozoa. It is treated by a series of experimental methods, the vigor of which is determined by the clinical and bacteriological symptoms. The early application and the intensity of the primary treatment exert a great influence upon the ultimate development of the disease.

Treatment by the intravenous or subcutaneous routes directed towards the elimination of the contagion is the method to be preferred in acute dysenteric attacks, in hepatic complications, and in some sub-acute or chronic forms[1].

Treatment by the mouth is preferable in certain chronic forms and very often it is the only method which results in the disappearance of the cysts.

Ipecacuanha in the form of Segond's pills or preferably in the form of double iodide capsules containing emetine and bismuth (according to Dumez's formula), and arsenic in the form of 1/10 gramme atoxyl pills or preferably of 1/10 gramme novarsenobenzol capsules.

On one day iodide of emetine in doses of 0.1 to 0.2 grammes; on the following day novarsenobenzol capsules of from 0.1 to 0.2 grammes and so on during the next 12 days.

Local treatment by enemas may be very helpful, although at times objectionable. Saline purgatives should be used. Adrenaline may give good results in amebic cases. It is well to nourish the patient as rapidly as possible, thus increasing his weight and powers of resistance.

The second meeting, April 20, at 10 A.M., was devoted to a report of the Trench Fever Commission of the Medical Research Committee of the American Red Cross in France. The results of the investigations carried out by the commission were presented in detail by the following members: Majors R. P. Strong and Homer F. Swift; Captains W. J. MacNeal, Walter Baetjer, and A. M. Pappenheimer, and Lieutenant A. D. Peacock.

A preliminary report of the work of the Commission has already appeared in the March number of the Medical Bulletin (N⁰ 5, p. 376). As a complete report of the Commission is being published by the American Red Cross Society, the addresses at this meeting are omitted from this report of the Session.

1. Every 4 days an intravenous injection of 3/10 grammes of novarsenobenzol is administered. On each of the intervening 3 days, emetine is injected in graduated doses of 4, 6, and 8 centigrammes. The first course of treatment includes from 6 to 10 arsenic injections alternated with emetine injections.

The Subject of the third meeting of the Session, April 20 at 2 P. M., was " Venereal Prophylaxis ".

Major Edward L. Keyes, Jr. M. R. C., read a paper entitled, " The Social Basis of the Campaign against Venereal Diseases in the American Expeditionary Forces. " He declared that the campaign against venereal disease in the American Army is being conducted upon a new and unique basis.

The speaker presented a chart recently received from the Office of the Surgeon General classifying " The Attack upon Venereal Disease " as follows :

PLAN OF THE ATTACK UPON VENEREAL DISEASE

I. Social measures to diminish sexual temptation

1. *Social Surroundings and Recreation.*

A. Within the Camp.

*a*) Social Facilities (Meeting Places).
*b*) Athletic Games and Recreation.
*c*) Education — Reading — Hobbies.
*d*) Places to meet Women Callers.
*e*) Music, Theatrical, and other Amusement.

A-1. Agencies :

*a*) Chaplains.
*b*) Y. M. C. A. War Work.
*c*) K of C.
*d*) Y. M. H. A.
*e*) Amer. Library Ass'n.
*f*) Commission on Training Camp Activities.
*g*) Y. W. C. A. Hostess Houses.
*h*) Post Exchanges.
*i*) Universities.

B. In Community.

*a*) Amusement.
*b*) Social Entertainment and Hospitality.
*c*) Information and Friendly Aid.

B-1. Agencies :

*a*) Playground and Recreation Association.
*b*) City Y. M. C. A.
*c*) Y. W. C. A.
*d*) Fraternal Orders.
*e*) Women's Clubs.
*f*) Commercial Associations.
*g*) Church.
*h*) School Authorities.
*i*) Travelers' Aid Society.
*k*) The Press.

2. *Suppression of Liquor Traffic.*

3. *Suppression of Prostitution.*

4. *Suppression of Indirect Temptations.*

*a*) Investigation and Surveillance.
*b*) Law Enforcement.

*c*) Prevention by Military Orders and Discipline.
*d*) Suppression of Vicious Amusements.
*e*) Censorsh p and Inspection.

Agencies:

*a*) Committee on Training Camp Activities.

*b*) S. G. O. Sanitary Corps.

*c*) Military Police.

*d*) Department of Justice.

*e*) Civil Police.

*f*) Civil Health Authorities.

*g*) State Committee on Councils of National Defense.

*h*) Soci l Hygiene Organizations.

*i*) The Press.

II. Educational Measures.

1. *Education of Soldiers.*

2. *Education of Civil Population.*

*a*) Lectures.

*a*-1 Special Instruction of Officers.

*b*) Literature.

*c*) Exhibits.

Agencies:

*a*) Committee on Training Camp Activities.

*b*) S. G. O. — Sanitary Corps.

*c*) Y. M. C. A.

*d*) Y. W. C. A.

*e*) Other Women's Organizations.

III. Prophylactic treatments.

1. *Instruction.*
*a*) Of Officers.
*b*) Of Men.
*c*) Of N. C. O. in charge of Station.
2. *Establishment of Prophylactic Stations.*
*a*) At Regimental Infirmaries.
*b*) In Civil Centers.
3. *Restrictions Upon Leaves of Absence and Passes.*
4. *Records and Statistics.*

IV. Medical Care.

1. *Military.*
A. Genito-Urinary Specialists.
*a*) Training of Medical Officers.
B. Laboratory Facilities.
C. Frequent Physical Inspection.
D. Standardized thorough Treatment.
2. *Civilian.*
A. Clinics.
*a*) U. S. Public Health Service.
*b*) American Red Cross.

The theory upon which the control of venereal disease is based may be divided as follows:

1. It is believed that the so-called sexual necessity of man is a relative and not an absolute condition, which depends upon habit and circumstance as much as upon hereditary tendency. It is well known that in the strenuous conditions of trench life, the sex impulse is greatly lessened, a fact which the men attribute to " saltpeter in the food ".

2. It is further believed that, although social, ethical, religious, patriotic, and other emotional influences are of great importance in dealing with the problem, they cannot always be relied upon to hold the evil in check. Therefore the campaign is based essentially upon hygiene, for it is believed that the sex impulse can be curbed by hygienic measures.

Reviewing the history of the control of venereal disease in the United States, the speaker said that in the Colonial period the social and religious conditions both in the cities and country districts were such as to prevent the problem from becoming an important consideration. In the nineteenth century, however, with the increase in city populations, a large prostitute class came gradually into existence. Little was done to control it, although when conditions grew so bad as to attract general attention, waves of protest and attempted suppression would for the time being give satisfaction to an outraged sense of decency.

Although sociologists and public officials, as a result of flagrant abuses, were in many instances prepared to concede the necessity for some form of license, as the only practicable compromise with the " oldest of the professions " and the " necessary evil " of masculine passion, nevertheless public opinion continued to regard any attempt at regulation as an incitement to sexual irregularity, a concession to vice, and a source of corruption in the police force.

While public opinion was still in this uncertain stage, Dr. Prince A. Morrow, of New York City, founded in 1903 the American Society of Sanitary and Moral Prophylaxis, which endeavored to inculcate the single standard of morality based upon the denial that the sexual necessity is imperative or necessary for good health and the maintenance of sexual vigor. It sought by education of the young to offset the forms of temptation arising from ignorance, and also to promote physical exercise and other forms of employment likely to diminish sexual craving. It also insisted upon the suppression of conditions encouraging vice in theatres, the press, dance-halls, saloons, etc. It sought also to awaken the public to the dangers of venereal diseases by a study of their epidemiology. Finally it insisted upon the repression of professional prostitution.

Twenty similar societies were quickly organized in other cities, many of which failed for want of determined and experienced leadership. From 1910 to 1915, however, the movement gained new impetus with the formation of the American Social Hygiene Association, founded by Dr. Morrow, of which Dr. Charles W. Eliot, President Emeritus of Harvard University, is now the honorary president. Its purposes are practically those of the earlier society, but the work is placed on a broader basis and receives much greater financial support. It endeavors to coordinate the work of local societies throughout the country, and to influence legislative control of the problem.

In 1913 the Bureau of Social Hygiene published a book by George J. Kneeland, entitled " Prostitution in New York City ", and in 1914 another by Abraham Flexner entitled " Prostitution in Europe ".

The book by Flexner was of importance in that it showed clearly by a careful investigation of the actual conditions in Europe that all attempts to control prostitution by registration, and so to protect the morals of the community, to discourage clandestine prostitution, and to reduce the incidence of venereal disease, had been of little avail. Such endeavors had for the most part failed because of the impossibility of registering the prostitute population. Although there are from 50,000 to 60,000 prostitutes in Paris, only 6,000 are registered. Out of 20,000 or 30,000 in Berlin, only 3,300 are registered. The facts are similar for the lesser cities.

Flexner attributes the impossibility of registration to the changing character of the prostitute population, especially because of a vast amount of child prostitution and of temporary prostitution. Even many of the confirmed prostitutes are crafty enough to evade all attempts at regulation. Furthermore, it cannot be shown that regulation has been effective in reducing to any great extent the incidence of venereal disease. The vast majority of prostitutes escape inspection altogether, and those who are inspected soon learn how to " cover up " the symptoms of disease, or, if this is impossible, they merely " disappear " and ply their trade in secret. Very little disease is actually revealed by the inspection, which, Flexner declares, " is only a pretext used to compel prostitutes suspected of criminal associations to report regularly to the police ". He continues : " There are physicians in Europe who believe that some form of medical inspection might be helpful; but none of rank who contend that it has ever yet been. When, therefore, medical inspection is urged on the ground that in Europe it is employed to reduce disease, you may confidently reply that regulation in Europe has most completely collapsed at precisely that point ".

Major Keyes, speaking next from the military point of view, declared that so far as the American Expeditionary Forces were concerned, the house of prostitution had served chiefly as a rallying point for vice, a club house for debauchery. He reviewed the army regulations so far enforced (G. O. 17, May 31, 1912, G. O. 31, September 12, 1912, and G. O. 45, Jan. 9, 1914[1]). They enjoin upon the medical officers of the Army the following duties :

1. Encouragement of physical exercise and of cleanly social intercourse and interesting mental occupation among the troops.
2. Instruction of the troops concerning the moral and physical dangers of intemperance and sexual promiscuity.
3. Venereal Prophylaxis.
4. Physical inspection for venereal disease twice a month.
5. Notation of dates of inspections on the monthly sanitary report.
6. Treatment of disease without hospitalization if possible.
7. Report of all cases of venereal disease.
8. Confinement of infectious cases.
9. Company officers and commanding officers are required to cooperate in bringing to trial those who fail to take prophylaxis and in ensuring the semi-monthly inspections.

The hygienic campaign was further extended by the " canteen law " to the sale of alcoholic liquors which were banished from all army posts.

To make the regulation regarding prophylaxis more effective, General Orders 6, 34, and 77, 1917, A. E. F., made all men contracting venereal disease subject to court martial, on the assumption that the prophylaxis was not taken at the right time, if at all, or that it was improperly administered.

In the United States the danger from the civil population was attacked by the Army Bill of May 18, 1917, prohibiting the sale of alcohol and the practice of prostitution within five-mile zones about camps. The good effects of these regulations and of the organization for their enforcement have induced all official and unofficial organizations interested in such matters, to support the strictest regulation of prostitution.

In France, no such condition exists. It is generally conceded that the satisfaction of the sexual impulse is imperative and that

1. These orders are given in full in an appendix to the Manual of the Division of Urology, American E. F.

the provision of regulated houses of prostitution is the most sanitary way of meeting this demand.

The speaker then met the argument in favor of regulated houses based on the assumption that venereal disease can in this way be controlled. He advanced proof that effective inspection and detection of disease among prostitutes was practically impossible even when they were in regulated houses. So few, however, were registered, and so many of those registered changed from place to place, that it was useless to claim that any kind of medical inspection could offset the venereal dangers. Furthermore, in the experience of the American army in France it had been clearly proven that under suppression of prostitution the incidence of venereal disease fell off to an amazing extent. During a period when prostitution was allowed, the incidence of disease was for the whites from 13 to 16 per thousand; and among the colored troops, from 18 to 108 per thousand. After the closing of the houses to the soldiers, the incidence of venereal disease among the whites dropped from 16 to 2 per thousand, and among the blacks from 108 to 11 per thousand. As the result of closing the houses, prophylactic treatments dropped off from 500 per thousand among the white troops to 130 per thousand, and among the colored troops from 150 to 51 per thousand.

Against the claim made that the repression of houses leads to clandestine prostitution and to the spread of venereal disease among the people, the speaker declared that such an effect had never been shown to occur. On the other hand, the police and the civil population are likely to respond to the moral force of a refusal to recognize prostitution. Very certainly, the temptations among soldiers were thereby greatly reduced.

The tripod supporting sexual licence was the following : youth, public opinion, and circumstance. It therefore behooved those who regarded prostitution as essentially a hygienic problem, to address themselves first to these sources of the evil, stamping out all discoverable aids in the spread of the infection.

Major GEORGE WALKER, M. R. C., said that the method of dealing with venereal disease in the army aimed, first, to prevent contact; and, second, to require prophylaxis after exposure.

Careful investigations at two of the ports of landing proved conclusively that houses were chiefly responsible for the incidence of veneral disease among American troops. Not uncommonly a woman in one of the houses might entertain as many as 80 men in the course of a day, and as many as 25 in a single afternoon.

Thus in the course of 10 days 60 women might entertain 50,000 of the troops

The situation was so bad that Major Young, after a visit, had an order issued to the effect that no permissions whatever should be granted to troops in transit at the ports. This measure had greatly improved the situation, and had reduced what was before not only a menacing but also a disgraceful state of affairs to a more or less normal condition which was easy to control. This order was made still more effective by forbidding all the men to be seen on the street in company with women. Thus the objection raised by many medical authorities, that the suppression of the houses would force the evil into the streets was effectively dealt with.

Furthermore it was found that these measures constituted no real privation to the men. No discontent resulted. It was clearly proven that normal, healthy-minded men did not need promiscuous intercourse to keep them contented.

With regard to prophylaxis, the requirements were that men should receive it within three hours after contact. Whenever this regulation was carried out, almost 100 o/o success was insured. This achievement was possible in spite of inadequate provisions for prophylaxis at the port stations. The speaker believed that with more careful study of the problem and with better equipment, an absolutely perfect result might be obtained. Although the study of the records was by no means a satisfactory basis for judgment, there could be little doubt that so far the results obtained by prophylactic methods had been extremely good. Out of a body of men which, on an average, numbered about 12,000, there was shown to be an incidence of only 0.9 per thousand. About 400 of the troops went to town weekly on permission, and many of them remained all night. This fact was largely responsible for the failures of prophylaxis, for it made treatment within ten or twelve hours practically impossible. The speaker expressed the hope that the all night permissions might be suppressed. He believed the present opposition to such suppression could be overcome, and that the results obtained would amply repay the effort.

The incidence of venereal disease in the navy was found to be greater than in the army, largely because permissions in the navy were for longer periods of time and also because the measures regarding prophylaxis were less exacting.

The speaker believed that American experience at the port towns had shown that gonorrhea and syphilis might be practically stamped out, provided that all leaves were cut to six hours and that thorough prophylaxis were required.

The chief difficulty encountered was among the negroes, who, at first, refused absolutely to take prophylaxis. Nevertheless, they were found to be the chief offenders both as regards the number of contacts and the incidence of venereal disease. Finally they were forced to take prophylaxis whether they wanted to or not, with the result that the incidence of disease fell to 1/5 its original figure.

A paper by Medecin-Major GOUGEROT, entitled "The Anti-Venereal Struggle outside the Army Zone" was read in translation by Major Rist.

*Part First* of the address was devoted to the anti-venereal struggle in military circles.

1. An attempt is made to educate the soldier, warning him against the dangers of venereal disease and giving him advice in matters of personal hygiene. To this end talks are given by the regimental doctors and pamphlets are distributed. An effort is also made to keep the soldier away from the prostitute.

2. Prophylaxis is recommended to the soldiers as a necessary precaution before and after intercourse. A semi-monthly health inspection is required of all soldiers. The writer stated that he had requested permission to try out the American system of compulsory prophylaxis in the IXth Region.

3. Every effort is made to detect venereal disease in its early stages. All cases are sent off at once to a venereal center or sub-center. The doctor who discovers a case is required to find out, if possible, the source of the contagion.

4. Venereal patients are treated in special hospitals and every effort is made to educate them with regard to subsequent care of themselves in all matters pertaining to their present infection or to the possibility of reinfection. All syphilitic cases are given, as a rule, a double dose of arsenic and mercury. In non-contagious cases, however, treatment is given in such a manner as not to interfere with the man's work.

5. When a patient is considered non-contagious he is allowed to return to his dépôt, but he is regularly inspected by the doctor, whose business it is to detect any return of contagious phenomena and to supervise courses of treatment. It has been found necessary to continue mercurial treatment for four or five years.

6. It has therefore been arranged to forward the patient's clinical record in sealed envelopes, in such a way as not to compromise him. Thus the military doctors to whom he may be confided from time to time will have all the necessary data for the continuation of treatment. It has been found highly important to follow up venereal cases, for otherwise recurrence is almost certain.

*Part Two* dealt with the conditions among foreign and colonial troops. The methods of education and treatment are practically the same as for the French troops. The educational pamphlets are translated into the various languages.

*Part Three* took up the problem in war factories. Here the conditions are especially difficult. Men and women, foreigners and colonials, are thrown together promiscuously under very discouraging conditions in powder factories and workshops. Such workers need the same kind of education as the soldiers. Prophylactic cabinets should be provided at the infirmaries and at the bathing establishments. Sanitary inspection should be required of all militarized workmen. At least the factory doctors should examine all persons coming for any kind of treatment with a view to detecting venereal cases. So far as possible, treatment should be carried out in such a manner as not to interfere with the patient's work. To render such measures more effective, a venereal wagon or bus, which may in a day visit several factories, has been proposed as the best means of providing for effective consultations.

*Part Four* dealt with the anti-venereal struggle among the civil population. In general the same methods are employed as in military circles. It is impossible, however, to impose upon a civil population any form of physical examination. Propaganda is distributed for educational purposes and clinics have been instituted in the form of " services annexes " in all towns and labor centers. These are anti-venereal dispensaries conducted by specialists. The " services annexes " ought to be centralized in such a fashion as to insure unity of action. In them should be centered the fight among civil populations against clandestine prostitution, and for the control of prostitutes in general.

It should be the business of the direction of the " annexes ", to hunt out the sources of contagions. He should endeavor by means of lectures, pamphlets, etc., to induce people to come voluntarily for treatment.

If possible the " services annexes " should be housed in separate buildings. They should be open for the treatment of all kinds of diseases, thus shielding venereal patients from publicity. Complete secrecy can further be assured by what the writer calls the " system of two degrees ", that is, an examination of all patients in a general reception room (first degree) and then the segregation of venereal patients thus discovered in a special room (second degree).

Although this plan providing for publicity and dispensaries has been put into operation in many places, it still meets with opposition in others. The writer therefore has for some time tried to secure legislation making such institutions obligatory wherever there is need of them.

*Part Five* made recommendations with regard to the control of prostitution. Since clandestine prostitutes are the most dangerous source of disease, men should be urged to frequent only the registered professional women. Every effort should then be made to insure complete hygienic control of the registered prostitutes. They should be required to carry official cards with photographs and thumb-prints. They should be required to undergo inspection at frequent intervals, but preferably not at definite appointed times. It should be the duty of the police to note the arrival of all new prostitutes and to keep a constant surveillance over their actions.

*Part Six* dealt with medical aspect of the control of prostitution. The venereal authorities must accept the general method of control in force in their region unless something better can be introduced, and then attempt to make improvements in the system from a sanitary point of view. The medical function should be carefully differentiated from the police activities. The policemen's duties should go no further than demanding the registration of prostitutes and the exhibition at regular intervals of cards certifying good health. In order that prostitutes may not be driven to evasion they should be allowed to present certificates of good health from reputable doctors of their own choosing if they do not wish to avail themselves of the public dispensaries. It is in general far better to induce registered prostitutes to present themselves voluntarily for inspection, than to harass them with too exacting regulations.

Incidentally everything possible should be done to reduce temptation and to suppress prostitution in the neighborhood of temporary centers like camps; also to suppress street-walking, procuring, etc. Care must be exercised that women suspected of having a disease should not be allowed to go to other places.

All found to be diseased should be compelled to go to a hospital pending radical and prolonged treatment.

Although such measures recommend themselves as sensible and easy to carry out, they meet with many and serious obstacles. The writer hopes that the educational campaign may be pushed with such earnestness that the civil population will see the dangers to which it is open, and be as eager to aid in the antivenereal fight as it has been in the campaigns against alcoholism and tuberculosis.

"Prophylaxis of Syphilis in the War Zone" was the subject of a paper by Médecin-Major SPILLMAN[1], Consulting Physician of the 8th Army, read in translation by Major Rist. As an indication of the rapid increase in the incidence of syphilis, the writer stated that in some units evacuation to venereological centers indicated that the incidence had nearly trebled during the year 1917.

The first step in combating the danger was an attempt to prevent the spread of the disease in the army by (1) early detection of cases in the primary or in the secondary stages, (2) evacuation of contagious cases to venereological centers, (3) treatment by specialists, and (4) the maintenance of treatment after the patient was returned to his unit.

Since the early detection of first stage cases was often too difficult for any one but a specialist with a well-equipped laboratory to make, the writer had arranged in his army for a specialist to paye periodical visits to the various units and so to make a definite diagnosis of all questionable cases.

The detection of cases in the secondary stage was greatly facilitated by the fortnightly required inspection. Doubtful cases could be sent for confirmation to the ambulance.

All contagious syphilitic cases discovered should at once be removed to the venereal centers; thus the danger of contagion in the unit, would be avoided and the use of the somewhat difficult modern methods of treatment be made practicable.

The writer insisted especially that medical records should be given to every patient as he returned to his unit, prescribing the necessary treatment to be followed in order to prevent the return of secondary symptoms. The unit doctors should keep their list of syphilitic cases strictly up to date, and see that the preventive treatment, such as, absorption of proto-iodine pills and intramuscular injections of mercury, was regularly carried out. The consulting specialist should supervise such treatment.

The writer recommended strongly a method of complete secrecy for the keeping of the man's record in the case of syphilis. This might be done by means of slightly disguised medical terms in the open records, and by the use of sealed envelopes to be given confidentially to the proper medical authorities. Thus all reluctance on the part of the individual men might be overcome.

1. See the *Medical Bulletin*, No. 1, November 1917, for a translation of reports presented at this meeting. Dr Spillmann's address appears on p. 64.

*Prophylaxis.* — Strict inspection before and after furloughs is an important means of protecting both the civil and military communities. Education by means of lectures and pamphlets and earnest recommendation of prophylactic methods before and after exposure are of prime importance. At present in the French army, the authorities could go no further, for owing to the present state of mind as to the delicacy of the subject, the enforcement of strict prophylaxis, as in some of the Allied armies, was out of the question. The speaker hoped, however, that such regulations might be introduced as the result of collaboration. At present the success of prophylaxis depended largely upon the skill and tact of the regimental doctors, some of whom had been very successful in winning the confidence of the men and in persuading them to adopt the proper preventive measures.

In the civil communities lay the chief danger to the soldier. In the army zone, rigorous control of prostitution and the confinement of the men to their service, made the danger comparatively slight. Out of 1987 cases of venereal disease in the writer's army from September 1916 to September 1917, 1466 were contracted out of the army zone and only 541 in the army zone. When men had the freedom of leave in civil communities the dangers were very great. These were less considerable among the registered prostitute class than among the much larger class of clandestine prostitutes, many of whom were not even commercial. Thus it was exceedingly difficult for any measures of control to be put into force.

Free prostitution, then, the writer contended, should be fought against by every possible means. He called attention to the recommendations made by him in a meeting of the chief surgeons of the venereological centers in July 1917 at Val de Grace, calling for the secret enrolment of unprofessional prostitutes in such a manner as not to endanger their standing in the community. Such clandestine prostitutes should be made to a show certificate of health at least once a week. Everything possible should be done to protect the professionnal prostitute who honestly submitted to the regulations, and every encouragement and facility should be offered to the secret prostitute to come to the "services annexes" for advice and treatment. The medical rather than the police authorities should take the initiative in all this service, inducing the women to come for treatment before they were forced to do so.

To make certain a uniform and efficient system, all such services among civilian populations should be under the control of the military specialists and coordinated in such a way as to produce maximum results in the whole campaign. Finally, every effort should be made to bring venereal disease into the open by removing as far as possible the stigma of shame, and by disseminating through pamphlets, lectures, and other forms of propaganda, all possible enlightenment concerning the dangers of venereal disease and the means of prophylaxis.

A paper entitled " Syphilis Prophylaxis " by Doctor GEORGE THIBIERGE, of the Hôpital St Louis, was read in translation by Major Rist. Dr. Thibierge, who was one of the first to call the attention of the French sanitary authorities to the need of syphilis prophylaxis, said that the long duration of the present war had so increased the incidence of the disease that it had now become a serious danger not only to the army but to the country at large. The sanitary condition of the country, in this respect, was largely the result of that of the troops. He expressed the desire to see everything done that might help in solving the problem, and he believed that the American medical forces might render material assistance.

The means of combating the evil, he declared, were : morality, hygiene, and medicine; also administrative and legislative measures.

The writer believed that the use of moral methods was better understood and further developed in America than in France. He praised American organizations which he had observed to render such practical service in keeping the soldiers from temptation. He was convinced also, he said, that religious principles did much to restrain men from sexual indulgence.

Medicine and hygiene, however, played a more direct part. This form of service began in the army with the teaching carried on by the regimental doctors especially for the young soldiers. The writer, however, entertained some doubts as to the prophylactic value of such instruction, for it had been shown among medical students that mere knowledge was not always sufficient to prevent them from running risks. It was a more practical part of the regimental doctor's duties to devise means of prophylaxis and to see that these means were actually employed by his men before and after intercourse. The writer stated that he was not altogether convinced that the prophylactic use of calomel ointment, as employed in the American army, produced any better results than soaping and the application of alcoholic solutions, and he hoped to have this question settled by what he could learn of the American experience.

A further duty of the army doctors was to inspect for cases of infection: these inspections were carried out fortnightly and before men went on furloughs. Thus many cases constituting a danger to the other men in the unit were brought to light.

*Prophylaxis by treatment*, the writer stated, was the present watchword among the sanitary authorities. The success of this method, however, depended at present upon inducing all syphilitics to come voluntarily to dispensaries or " annexes " for treatment. It was obviously impossible to accomplish this in the case of clandestine prostitutes, degenerates, etc. *Prophylaxis by treatment* therefore, was not effective unless it were accompanied by rigorous administrative surveillance of prostitution. Whenever such surveillance had been vigorously enforced, it had given good results, but unfortunately in many cases objections to such control were made from so many sources that local administrations had at last taken refuge in the comfortable doctrine of *prophylaxis by treatment* on a voluntary basis.

It was now time for rigorous measures to be taken through proper legislative channels to control prostitution, providing for internment of known syphilitics, and perhaps even compelling the confession of venereal disease.

Such legislation ought also to provide for the abolishion of all quack establishments for the cure of venereal disease, as had recently been accomplished by the British authorities.

Major Hugh H. Young, M. R. C., Director of the Division of Urology, A. E. F., read a paper entitled, " Success of the Campaign for Combating Venereal Disease, in the American E. F. ".

The Campaign against Venereal Disease in the A.E.F. consists briefly of the following:

1. *Social Hygiene.* — For the purpose of minimizing the number of sexual contacts.

2. *Prophylactic Treatment.* To prevent the development of venereal disease after sexual contact.

3. *Physical Inspection of Troops.* To detect venereal disease with the object of instituting prompt and efficient treatment in order to reduce the number of days sick; and of restricting leave in order to combat the spread of infection.

4. *Repression of Prostitution.*

5. *The Reporting of Sources of Infection* and Dispensary treatment of Civil Population.

6. *Enforcement of Laws Relating to Alcoholism.*

7. *Court Martial for Venereal Disease.*

8. *Treatment of Venereal Disease with the Organizations* in order to reduce the loss of effective strength and to avoid making venereal disease an excuse for escaping duty.

The forceful general orders 6, 34, and 77, A.E.F., 1918, have furnished the basis for a very active and radical campaign.

The wonderful laws passed by congress for the protection and enlightenment of our troops in America have been considerably supplemented by very vigorous and far-reaching orders in France.

G.O. 6., 1917, A.E.F., requiring that *all* men with venereal disease be tried by court martial and punished by loss of pay has largely put an end to efforts to escape punishment by falsifications on the prophylactic records.

This order, which was one of the first General Orders issued after arriving in France, dealt with the frequency and danger of venereal diseases, and dwelt upon the responsibility of company commanders and medical officers towards their men. The intense interest displayed in the physical condition, health, and morals of his men by the Commander-in-Chief as shown in this and subsequent general orders, has had a wonderful effect in creating a fine *esprit de corps.* Organizations now take pride in declaring that " We have a clean bunch here ".

The radical provision requiring that all men contracting venereal disease be court-martialed and punished by loss of pay, a punishment which had previously been often avoided by taking a prophylaxis after the disease had started and getting the name on the register — has not only done away with the reason for this deception, but has forced men to abstain or to make every effort to prevent disease by taking early prophylaxis to escape the court-martial.

G. O. 34 which again urged sexual continence and high moral standards of living, and required that men, arriving in Paris and other cities, have papers viséd, be given warning as to the danger and prevalence of venereal disease, and be required to reside in barracks or hotels designated by the Provost Marshal where prophylactic stations have been organized, has had a splendid effect in prevention of debauchery and venereal disease.

The provisions of G. O. 34 promulgating a plan for treatment of venereal patients without evacuation to hospitals, but at dispensaries where they would remain on duty status, continuing at work drill, has had a wonderful effect in preventing loss of effectives and in doing away with the voluntary acquiring of venereal diseases to avoid military service, as has often happened in other armies.

At the same time, the possibility of early radical and consecutive intravenous treatment has been secured for the first time in any army in the field.

As a result, instead of now filling three one-thousand-bed hospitals with venereal patients as had been expected by this time and planned for in the U. S., *there are no venereal hospitals*, and only about three hundred beds occupied by venereal cases, and these largely in regimental and field infirmaries.

G. O. 77 which dealt radically with base posts, prohibiting shore leave to soldiers still on transports, restricting soldiers to camps, placing the saloons and houses of prostitution out of bounds, and again directing officers to assist in making venereal prophylaxis effective has had a really marvellous effect. A previously riotous seaport city has been transformed; drunkenness there has almost disappeared, licentiousness has been rudely curbed and the venereal infections have been reduced to a minimum.

The order requiring that the venereal status of an organization be filed at G. H. Q. with the personal records of the Commander to be "used as a basis in determining the Commander's efficiency and the suitability of his continuing in Command" has been of immense value in awakening a sense of responsibility in the health and hygiene and effectiveness of the troops of his command. This is one of the most powerful army sanitary orders ever issued.

*Sexual Continence.* In various general orders and circulars, sexual continence is urged upon the troops. Is this at all possible in armies? There are many who scoff at the idea. What do our statistics show in this regard? It is, of course, impossible to get accurate statistics upon these delicate subjects. Even well-disciplined soldiers, although ordered to take venereal prophylaxis after each sexual intercourse, are not infallible in obeying these orders, but we believe that the great efforts which have been made in the A. E. F. to make troops realize the danger of venereal disease has had the effect of making the great majority take venereal prophylaxis after sexual indulgence. Where prophylaxis is not taken the venereal rate becomes high. We may, therefore, utilize the statistics of the prophylactic stations as indicating in a fair way the amount of sexual congress among the various organizations. The following table taken from the troops of one Division who had been about seven weeks in France, is exhibited as an argument for the possibility of having almost complete sexual continence among fairly large numbers of soldiers.

TABLE I

| Organization | Strength | Time in France | Venereal prophylaxis taken | Venereal Disease acquired |
|---|---|---|---|---|
| Amb. Co. . . . | 115 | 6 weeks | 0 | 0 |
| H. Q. Troops. . | 330 | 4 " | 0 | 0 |
| M. G. C. . . . | 582 | 6 " | 0 | 0 |
| Bn. Inf. . . . | 1,367 | 8 " | 6 | 0 |
| Amb. Co . . . . | 122 | 6 " | 2 | 0 |
| Amb. Bo . . . | 123 | 8 " | 0 | 0 |
| Eng . . . . . . | 515 | 8 " | 16 | 0 |
| M. G. C . . . . | 570 | 8 " | 17 | 0 |
| S. C. . . . . . | 270 | 8 " | 4 | 1 |
| Reg. Inf . . . . | 3,267 | 8 " | 11 | 0 |
| Totals . . . . . | 7,401 | av. 7 weeks | 56 | 1 |

Analyzing this chart, we see that in one infantry battalion of 1367 men who were eight weeks in France, only six prophylactic treatments were taken. The fact that four careful inspections failed to reveal any venereal disease is given as evidence that the prophylactic records are probably fairly correct. Three venereal cases which are present were with the organization on arrival in France.

Another notable case is that of the infantry regiment — the last on the list — 3267 men which had been two months in France, during which time only 11 prophylactic treatments were taken and no venereal disease acquired. Sixteen cases of venereal disease, all old cases present when the regiment landed in France, was the sum total of venereal disease in the regiment, a venereal rate of 5 per 1000 or one half of one per cent. The total number of men in this table was 7401, the average length of stay in France 7 weeks; the total number of venereal prophylaxes 56. Among these 7000 men, during a period of almost two months, only one new case of venereal disease was discovered after four careful physical examinations were made. This record, of course, is remarkably good — probably better than that of the Army as a whole, but these organizations are cited as evidence that sexual continence can be maintained by large bodies of troops for a considerable period of time.

This has, however, already been demonstrated by the French and British armies, whose troops have remained for long periods (varying from four to twelve months) at the front where there are no women, and where sexual continence is the rule per force of circumstances. No one has ever suggested that these troops were not in fine fighting trim, or that their morale had been diminished by their excellent morals. One of the best preparations for the zone of the front in which troops have to follow enforced continence, is to have troops in training, habituated to getting along without sexual indulgences. This long drawn out war of the trenches has exploded another old-time fallacy, i. e., that the soldier must be a libertine in order to be a fighter.

*Efficacy of Prophylaxis.* The Army statistics for 16 years have effectively demonstrated the wonderful results which can be obtained by prophylactic treatment in preventing venereal disease. One of the most careful studies on the subject is that contained in a recent report by Riggs, from which the following table is taken :

*Number of prophylatic treatments, failures and percentages for each hour after exposure*

| Hours subsequent to exposure | Number of treatments | Number of Infections | Per cent. of Infections |
|---|---|---|---|
| 1. | 1180 | 1 | 0.08 |
| 2. | 1172 | 7 | 0.59 |
| 3. | 521 | 4 | 0.77 |
| 4. | 330 | 2 | 0.61. |
| 5. | 199 | 3 | 1.57 |
| 6. | 321 | 5 | 1.58. |
| 7. | 277 | 6 | 2.27. |
| 8. | 390 | 16. | 4.22. |
| 9. | 283 | 10 | 3.62. |
| 10. | 214 | 11 | 5.14. |
| More than 10 | 216 | 16 | 7.40 |
| Total . . . . | 5103 | 81 | 1.58. |

" There were 1180 treatments during the first hour which were followed by a single infection. This infection was carefully investigated and there is considerable doubt as to whether it was genuine or not. The disease was diagnosed as chancroid, and was cured in two days.

" There are no statistics from which we may deduce the normal percentage of venereal infections that may be expected to follow illicit sexual intercourse when prophylactic means are not employed. In the above table, those who took prophylaxis later than ten hours after exposure give a rate of infection of 7.4 per cent. and applying this percentage to the 6746 recorded exposures gives an expectancy of 499 venereal infections. As a matter of fact, there were only 127 subsequent infections, so it may be assumed that the difference 372 represents the least number of venereal infections which were prevented by the 6746 treatments. It is unlikely that the venereal situation in this community would have been better had the 6746 prophylactic treatments been withheld, and these 372 venereal infections been permitted to take place ".

Study of the Medical records of the A. E. F. amply testifies to the accuracy of these assertions.

In one of the base areas over a period of three months, among troops varying from 7,272 to 16,301, 9,129 venereal prophylaxes were given (about one to every four men per month, but many offenders were each responsible for several V. P. so that the actual number of men having intercourse was much less than 1 in 4). Among these troops there were during this period 374 venereal diseases discovered at the bi-weekly inspections. In 144 of these no venereal prophylaxis had been taken, leaving 230 cases of venereal disease in which the prophylactic treatment had been given, showing failures in 2.5 o/o. Many of these troops were grouped in small units in which medical organization and supervision were difficult, so that the records are not as good as those obtained elsewhere.

The record of 8000 troops, a part of one of the divisions which arrived during the summer at one of the ports in France and proceeded, after a short stay, to one of the training areas in Eastern France in which there were numerous villages, with a fairly large population, among which they were billeted, showed that there had been 2,363 venereal prophylaxes taken at the port of debarkation (where houses of prostitution then flourished) and 130 in the training camp. During the month that these troops had been in France 28 cases of Gonorrhoea, 8 cases of Chancroid and no case of syphilis had developed. My records do not show how many of these men with venereal disease had taken prophylactic treatment, but it is fair to assume that the majority had not taken it; but even if all are reckoned as failures of venereal prophylaxis to prevent disease, the percentage of failure for the 2,493 prophylaxes taken would be 1.4 o/o. The following chart, in which accurate records have been kept, shows what can be obtained by prophylactic treatment even when the number of sexual contacts is considerable.

TABLE III

| Organization | Strength | Prophylaxes taken | Venereal disease cases | Failures of Prophylaxis |
|---|---|---|---|---|
| Eng. . . . . . . | 987 | 1328 | 20 | 1.5 o/o |
| F. Art. . . . . . | 855 | 248 | 6 | 1.4 " |
| Eng. . . . . . . | 982 | 545 | 9 | 1.6 " |
| F. Art. . . . . . | 834 | 283 | 3. | 1.0 " |
| B. Hosp. . . . . | 213 | 274 | 2. | 0.7 " |
| Total . . . . . . | 3995 | 2727 | 36 | 1.4 " |

The general prophylactic efficiency rate of 1.4 o/o is good, but indicates that an average of 4 hours elapsed according to Rigg's table. The last unit with a rate of 0.7 o/o shows what ought to be accomplished with prophylaxis.

The following records from some of the smaller organizations are of interest :

One battalion — of Infantry at seaport base for two months on police duty, during which time ample chance for sexual congress was present, as shown by 392 venereal prophylaxes taken, — had only two new cases of venereal disease during this time.

A Headquarters Troop with a present strength of 460, in France six months, had in its personnel only one new case of venereal disease, and in this no venereal prophylaxis had been taken. In fourteen units with a personnel of from 150 to 200 each and totaling about 2,500 men, there were in the month of February only 4 cases of venereal disease (and confined to three of these units in which 216 prophylactic treatments had been given (1.8 o/o failure). In the other 11 units 572 prophylaxes were given and there were no infections. Four Base Hospital units, with an average personnel of 200, had, in six months, no venereal disease.

Among the troops in the field the rate per 100,000 men was 1502 prophylaxes and 66 cases of venereal disease, showing a failure in about 4 o/o of the cases. These troops were frequently on the move, and often remained for a day or two in small towns or cities where it was difficult or impossible to arrange prophylactic stations, and in which abundant chance for intercourse was at hand. This accounts for the higher percentage of failures.

In September 1917, before the propaganda against venereal disease and methods of prophylaxis and treatment had been thoroughly organized, the monthly rate of new cases was 4.2 per thousand, whereas in January 1918 the month's venereal rate had dropped to 2.5 per thousand, and in March to 2 per thousand certainly a splendid record for the A. E. F.

The *relation between high venereal rate and the failure to take prophylactic treatment is very definitely shown by many of our records.* Thus in two artillery regiments with a strength of about 850 men, and with the same number of prophylaxes taken during a given period, the venereal rate in one was 79 and in the other 25, as shown in the following chart :

| Organization | Strength | No. of V. P | Av. per man | V.D. after V. P. | Failure | V.D. and no. V.P. | Total | Rate per 1000 |
|---|---|---|---|---|---|---|---|---|
| — X | 855 | 478 | 0.56 | 7 | 1.4 | 7. | 14 | 70 |
| — Y | 834 | 450 | 0.52 | 5 | 1. | 0. | 5 | 25 |

As will be noticed here, the average venereal prophylaxes taken *per man* was about the same — 0.5 but in the first it was not so successful, so that 1.4 o/o as compared to 1 o/o, but the principal difference arose from the fact that in the first regiment there were 7 cases of venereal disease in which no prophylaxis had been taken, and in the other no cases of venereal disease from failure to take prophylaxis, thus making the total venereal disease in one regiment 14 and the other 5, and the venereal rate 70 per thousand in one and in the other 25 per thousand for the whole year — a remarkably low rate.

The table given below is of two organizations of nearly equal strength — one well regulated, the other a motor-truck company " running wild "; the period is for 3 1/2 months. In organiz-

| Organiz-ation | Strength | V. P. | Av. per man | V.D. after V.P. | Failure of V.P. | V.D. and no. V.P. | Total V.D. | Annual rate p. 1000 |
|---|---|---|---|---|---|---|---|---|
| A. | 212 | 240 | 1.1 | 2 | .8 | 1. | .3 | 50 |
| B. | 206 | 350 | 1.7 | 12. | 4.0 | 2. | 14 | 328. |

ation B. the V. P. taken were 40 o/o greater and the failures five times as great. There were also twice as many cases of V. D. in which no prophylaxes had been taken, and together they gave the startling difference in annual rate per 1,000 (50 and 328).

Inquiry showed that in B. the prophylaxes were taken much later than with A.

In two infantry battalions a tremendous difference in the V. D. rate was caused by the fact that one commander gave frequent all night leaves — the men taking their prophylaxes on return next day. The rate was three times as great as in the other in which men were required to be back at night.

Another comparison which brings out the fact that the venereal rate is proportional to sexual contacts is shown in the records of the two organizations given below, in both of which the venereal prophylaxis was about equally successful (1.6 o/o and 1.4 o/o).

| Organiz-ation | Strength | V. P. | Av. per man | A.V. after V.P. | Failure of V.P. | V.D. and no V.P. | Total V.D. | Rate p. 1000 |
|---|---|---|---|---|---|---|---|---|
| — Eng. . | 982 | 559 | 0.6 | 9. | 1.6 o/o | 3 | 12 | 49 |
| — Eng. . | 987 | 1494 | 1.5 | 22 | 1.4 o/o | 6 | 28 | 109 |

The number of men in each organization is seen to be about the same, but the average coitus per man as shown by the prophylaxes taken was almost three times as great in one organization, and the venereal rate was also nearly three times as great. These figures show conclusively that even if the venereal prophylactic methods are wonderfully successful and only fail in a small percentage of the cases (1.5 o/o), it is extremely important to bend every effort to minimize the amount of sexual contacts, and thus reduce the possibility of infections. Our statistics prove conclusively that this is accomplished best by putting the houses of prostitution out of bounds, and thus reducing the facilities for obtaining sexual intercourse among the soldiers, as shown by the following record from a base front.

CHART IV

| Whites | Month | Strength | V. P. | o/o per man | V.D. cases | V. D. rate p. 1000 | |
|---|---|---|---|---|---|---|---|
| Houses open to soldiers | Aug. | 4571 | 1669 | 43 | 72 | 16 | V. P. not taken early; rate high. |
| | Sept. | 9471 | 3392 | 36 | 124 | 13 | |
| | Oct. | 3966 | 2074 | 52 | 67 | 16 | |
| Houses out of bounds | Nov. | 7107 | 885 | 12 | 81 | 10 | First half of Nov. not closed. |
| | Dec. | 4281 | 539 | 12 | 44 | 10 | |
| | Jan. | 3777 | 523 | 13 | 8 | 2 | |
| **Negroes** | | | | | | | |
| Houses | Aug. | 430 | 14 | 3 | 20 | 19 | It was difficult to get negroes to take V. P. |
| | Sept. | 411 | — | 0 | 21 | 51 | |
| | Oct. | 607 | 91 | 15 | 66 | 109 | |
| Houses out of bounds | Nov. | 1807 | 398 | 22 | 142 | 103 | First half of Nov. not closed. |
| | Dec. | 2830 | 111 | 4 | 59 | 21 | |
| | Jan. | 5613 | 289 | 5 | 62 | 11 | |

During the months of August, September, October and the first half of November, the houses of prostitution flourished and were filled with soldiers. On November 15 rigid orders were issued placing these houses out of bounds, and the immediate result was a great reduction in the number of sexual contacts, as shown by the number of venereal prophylaxes taken. As a result there was a steady decline in venereal infections, and the monthly rate per thousand, which in October reached 16.8 dropped in January to 2.1 among the white troops. During the same period there was an even more startling drop in the venereal infections among the negro laborers, the percentage dropping from 108.7 per thousand per month to 11 per thousand. No statistics could speak more eloquently for the doctrine of closing the houses of prostitution. Our studies showed numerous infections coming from houses " inspected " three times a week.

A recent report by a Consulting Urologist is also of interest. " I have noticed in the base sections that very few of the enlisted men can speak French, and many of them for this reason refrain from speaking to a girl on the street, and, if they do, many even then are afraid to go to a strange place with her on account of lack of a clear understanding. These soldiers, on the other hand, find no difficulty in going to a house of prostitution where an interpreter is always at hand, and where little talking is necessary. The houses of prostitution are prepared to do a wholesale business in sexual commerce, while the clandestine prostitute must of necessity be restricted in the amount she can accomplish in a given time. As an example, I cite the instance of the 60 prostitutes in houses at — having had during ten days in September 15000 sex relations." With every facility afforded for obtaining sexual intercourse, even though the venereal prophylaxes are always taken, and the rate of failure for prophylaxis is small, if the number of sexual contacts greatly increases, the venereal rate must also increase. The necessity, therefore, of restricting sexual contacts is self-evident, and the need of putting the houses of prostitution out of bounds is imperative.

*Venereal Prophylaxis Stations.* — The following report from a Urologist concerning one of the venereal prophylactic stations located in a city of about 100,000 population is given. In this city, during one month, 799 prophylactic treatments were given American soldiers. These men were carefully questioned, and it was found that of the 799 men, 761 had been to houses of prostitution and 33 had been with clandestine prostitutes. In the case of 5 no record was obtained. In the same city, a study of the venereal cases during two months — 27 cases of venereal disease in acute stages — showed the following sources of infection : — U. S. 9. Paris 1, Besançon 1, Saint-Nazaire 1, a total of 12 cases which had not been acquired in the city. Nineteen of the 27 patients had received no prophylactic treatment. Only three claimed to have received the treatment within three hours. There was a marked variation in patronage at the prophylactic stations according to the days of the week. On Monday, Tuesday, Wednesday, Thursday, and Friday of each week, there were very few prophylaxes given. On Saturday the number increased, but by far the greater number were given on Sunday.

*Records of the Paris Venereal Dispensary.*

| | |
|---|---|
| From April 1 to 20, V. D. cases | 42 |
| No. of V. P. taken among these | 25 |
| V. P. administered to self | 2 |
| Uncertain (records elsewhere) | 9 |
| V. P. after all night | 2 |
| V. P. in three hours | 1 |
| " " two " | 2 |
| " " one " | 1 |

*Analysis :*

| | |
|---|---|
| Failure after three hours or less. . . . . . . . | 4 |
| Total number of treatments given. . . . . . . | 785 |
| o/o of failure of V. P . . . . . . . . . . . . . | 0.5 o/o |

The great cause of venereal infection has been the baneful effects of houses of prostitution, and unbridled street solicitation, which are responsible for most of the contacts, and the failure to take prophylaxis early. Efforts now being made to enforce the wise and comprehensive provisions of G.O. 6, 34, and 77 are bearing fruit splendidly.

Those organizations which have a high venereal rate generally have inefficient line and medical officers. It is hoped that the three provisions of G.O. 77 will bring them to book.

The following comparison between the U. S. Army in 1916 and the A. E. F. in 1918 from the report of April 8, 1918 is as follows : — The U. S. army 1916 venereal non-effective rate per 1000 was 5.62, while in the A. E. F. it was 2.13.

The venereal rate just reported for last week is even better, being exactly 2 per thousand.

The figures speak for themselves, and no arguments are necessary to show the splendid record of the A.E.F., and the efficiency of the extensive programme which is being carried out to combat venereal disease, in which General Pershing and General Bradley have taken such great interest.

A Report was read by title from the Médecin-Major (1re Classe) GAUDUCHEAU of the Colonial Troops. He recommends an individual prophylactic ointment consisting of :

| | | |
|---|---|---|
| Thymol . . . . . . . . . . . . . . . . . . | 2.50 | grs. |
| Calomel. . . . . . . . . . . . . . . . . . | 15 | " |
| Vaseline . . . . . . . . . . . . . . . . . . | 10 | " |
| Lanoline. . . . . . . . . . . . . . . . . . | 80 | " |

The addition of thymol to the original formula of Metchnikoff, the writer considers an important step in advance, because it produces an immediate antiseptic action. Even in greasy and albuminous media (for instance the yolk of an egg) its antiseptic action against the vibrion cholérique was unimpaired to a limit of 1 in 2000. Since in the formula thymol is in the proportion of 2.50 to 100 or 1 in 40, it is highly likely that organisms which are so slightly resistant to antiseptics as the gonococcus and the treponema are immediately killed in the ointment. It does not, however, produce any noticeable irritation.

It is contained in a tube supplied with a glass or wooden cannula with lateral openings, by means of which the ointment may be introduced directly into the urethra without any more elaborate apparatus. It should also be spread on all other mucous surfaces and cutaneous excoriations. Thus complete prophylaxis is afforded against gonorrhea, syphilis, and chancroids, far more effective, in the writer's opinion, than any form of police restriction or moral sanitation in preventing the incidence of venereal disease.

He insists, also, upon prophylaxis for old cases of syphilis, for he is convinced that although a person who has once contracted syphilis may not have another chancre as a result of contamination, reinfection manifesting itself in secondary and tertiary lesions is entirely possible, accordingly as the normal body reaction is or is not modified by the earlier infection.

The writer is inclined to believe that the various manifestations in syphilitic lesions are not so much due to changes in the activity of the infecting organisms as to the changes in the physiological condition of the body. He is even inclined to believe that many of the very late manifestations of syphilis may be due to reinfection and not represent a stage of the original disease. He believes that syphilis may therefore not have so long a course as has sometimes been supposed.

Even if the original strain of organisms persists, all risk of reinfection should nevertheless be avoided, and hence prophylaxis should be practised by those who consider themselves immune because of previous infections.

The paper on venereal prophylaxis by Captain H. L. WALKER, R.A.M.C., has been not received.

The following abstract of a paper by Captain Moss, M.R.C., was delayed in transmission and arrived too late for publication in the April number of the Bulletin with the papers for the March meeting of the Research Society it was entitled " Blood groups and the selection of donors for transfusions. "

Captain Moss stated that it was a familiar observation that the serum of one animal species may agglutinate or hemolyze the red blood corpuscles of another species. This characteristic alone is sufficient to determine a distinct biological difference between the species.

It seems a somewhat remarkable fact that such a difference may occur within a species but such is the case in man. The serum of one person may agglutinate the corpuscles of another person and, less frequently, may hemolyze them. Such reactions are known as isoagglutination and isohemolysis[1].

The group of a person is rarely established in his blood at birth but usually becomes so within the first few months of life and, once established, it apparently never changes.

From the above tabulation of the incidence of the four groups it is seen that approximately 95 per cent. of human serum contains agglutinin for the corpuscles of certain other individuals. The ability of the serum to hemolyze the corpuscles of other persons is met with much less frequently but is by no means rare. It is found that the serum of about 25 per cent. of humans is capable of hemolyzing the corpuscles of some other persons.

When isohemolysins are present they are found to act according to the same laws as those governing isoagglutination, at least so far as the groups are concerned. For example, the serum of Group I, which contains no isoagglutinin never contains isohemolysin. The corpuscles of Group I, which are always agglutinated by the serum of Groups II, III, and IV, may be hemolyzed by the serum of Groups II, III, and IV provided the latter groups contain isohemolysin. The serum of Group II which always agglutinates the corpuscles of Groups I and II may hemolyze the corpuscles of the same groups if it contains isohemolysin, and the corpuscles of Group II may be hemolyzed by the serum of Groups III and IV if the latter groups contain isohemolysin. The isohemolytic relations for Groups III and IV may be stated in a similar way.

The isoagglutination relations between the four groups may be

1. Based upon the isoagglutination reaction, human beings may be divided into four groups which are called isoagglutinin or, less specifically, blood groups.

The classification may be conveniently stated as follows :

| | |
|---|---|
| *Group I.* | Serum agglutinates corpuscles of no person. |
| | Corpuscles are agglutinated by serum of Groups II, III, IV. |
| *Group II.* | Serum agglutinates corpuscles of Groups I and III. |
| | Corpuscles are agglutinated by serum of Groups III and IV. |
| *Group III.* | Serum agglutinates corpuscles of Groups I and II. |
| | Corpuscles are agglutinated by serum of Groups II and IV. |
| *Group IV.* | Serum agglutinates corpuscles of Groups I and III. |
| | Corpuscles are agglutinated by serum of no person. |

Groups I and III are rare groups ; groups II and IV are common. The relative incidence of the four groups is about as follows :

| | | |
|---|---|---|
| Group I . . . . . . . . . . . . . . . . . . . . . | 5 | per cent. |
| Group II . . . . . . . . . . . . . . . . . . . . | 40 | " |
| Group III. . . . . . . . . . . . . . . . . . . . | 10 | " |
| Group IV. . . . . . . . . . . . . . . . . . . . | 45 | " |

shown graphically by the following schema, in which no agglutination is represented by o and agglutination by +

| Serum | | Corpuscle Groups. I | II | III | IV |
|---|---|---|---|---|---|
| Group | I . . . | o | o | o | o |
| " | II . . . | + | o | + | o |
| " | III. . . | + | + | o | o |
| " | IV. . . | + | + | + | o |

Reference to the above schema shows that the group to which an individual belongs may be determined by testing his corpuscles against each of the four known sera or by testing his serum against corpuscles from each of the four known groups. The results may be represented as follows, letting × represent the corpuscles of the individual whose group is to be determined.

| | | Serum Groups. I | II | III | IV | | | |
|---|---|---|---|---|---|---|---|---|
| Corps. | × | o | + | + | + | = | Group | I |
| " | × | o | o | + | + | = | " | II |
| " | × | o | + | o | + | = | " | III |
| " | × | o | o | o | o | = | " | IV |

It is easy to formulate the results if the serum of × is tested against corpuscles of the four known groups.

Examination of the middle two vertical columns in the above schema shows that the group of an individual may be determined by testing his corpuscles against Group II and III sera, moreover it is possible to determine the group of an individual by testing both his serum and corpuscles against the serum and corpuscles of a single known group provided the known group is either a II or III.

Technique of group determination : Given known Group II and III serum the determination of the group to which any individual belongs is easy, and various simple procedures have been devised, two of which will be described.

1st *Method. Microscopic Test.*

Allow a drop of blood from the person whose group is to be determined to fall into a test-tube containing about 3 cc. normal salt solution. Secure a suspension of the corpuscles by immediately shaking or rotating the tube. Take two hollow-ground slides (or one slide with two depressions), mark one II and the other III. Place on each of two cover slips a very small drop of corpuscle suspension and to one add an equal sized drop of Group II serum. Mix the serum and corpuscles with a platinum loop or with a capillary glass stirring rod. Invert the cover slip over the hollow-ground slide marked II.

To the corpuscles on the other cover slip add a very small drop of Group III serum, mix and invert over the hollow-ground slide marked II.

Observe immediately under the microscope to see that a uniform suspension of corpuscles has been obtained (agglutination may begin to take place in one to two minutes). Examine again at the end of an hour. If no agglutination has taken place by this time, at ordinary temperatures the result may be considered negative. Warming up to 37 1/2° C. and slight agitation hastens agglutination.

*Four possible results may be observed.*

1. Corpuscles on both slides agglutinated (Group I corpuscles).
2. Corpuscles on slide II not agglutinated. Corpuscles on Slide III agglutinated (Group II corpuscles).
3. Corpuscles on Slide II agglutinated, Corpuscles on Slide III not agglutinated (Group III corpuscles).
4. Corpuscles on neither slide agglutinated (Group IV corpuscles).

It sometimes happens that the corpuscles as they are agglutinated are hemolyzed so that if the observation is not made until after the lapse of an hour or more no corpuscles are seen. This result should be considered the same as agglutination in determining the group of the corpuscles tested.

*2nd Method. Macroscopic Test.*

Captain Beth Vincent, U. S. R., has devised a very simple and practical macroscopic test. To the known Group II and III serum 1.5 o/o sodium citrate is added. An ordinary microscope slide is used, one end of which is marked II, the other end III. A small drop of Group II serum is placed on the slide toward the end of the slide marked II and a similar drop of Group III serum is placed on the slide toward the other end. The ear or finger of the person whose group is to be determined is pricked and a very small drop of blood is taken on the point of an ordinary pocket knife and mixed with the drop of Group II serum. The blade of the knife is then carefully wiped dry and the procedure is repeated for the Group III serum. If agglutination is going to occur, it is usually apparent within five minutes but the result should not be called negative until fifteen minutes or half an hour have elapsed. Agglutination is promoted by gently agitating the slide.

The known Group II and III sera for both the above methods may be kept on hand in the laboratory and if kept sterile retain their agglutinins for six months or a year. Even a moderate degree of contamination may not interfere with the tests.

*The selection of donors for transfusions.*

It is now generally recognized that in the selection of donors for transfusions the blood groups should be taken into consideration. Our knowledge is incomplete concerning the causes of the untoward results which occasionally occur when the blood groups are disregarded in transfusion. These results may be mild or grave and are manifested by one or more of the following symptoms : cyanosis, respiratory distress, chill, fever, icterus, hemoglobinuria, anuria and sometimes death. The onset is usually immediate and in the mild cases the symptoms may be over in half an hour. In the moderately severe and severe cases some of the symptoms may persist for 24 to 48 hours and the patient completely recover from what appears to be an extremely critical condition. In the fatal cases death usually supervenes quickly and rarely is delayed beyond one or two days.

It is possible, though I think not proven, that isoagglutination may play a part in the production of some of the symptoms and it is certain that isohemolysis does.

One may naturally ask the question : Why, if the symptoms are due to isohemolysis, were they not more frequently noted in the days when transfusions were done without reference to the groups?

Assuming that the incidence of the various groups are as given above and that donors and recipients are taken at random it is possible by the laws of chance to calculate the relative frequency with which the various possible combinations of groups will be met. The results are shown in tabular form below.

| Group | I | II | III | IV | |
|---|---|---|---|---|---|
| " I | .25 | 2.00 | .50 | 2.25 | 5 o/o |
| " II | 2.00 | 16.00 | 4.00 | 18.00 | 40 o/o |
| " III | .50 | 4.00 | 1.00 | 4.50 | 10 o/o |
| " IV | 2.25 | 18.00 | 4.50 | 20.25 | 45 o/o |
| | 5.00 | 40.00 | 10.00 | 45.00 | |

The only combinations in which the possibility of agglutination (and hemolysis) does not exist is where donor and recipient are members of the same group. The incidence of such combinations are as follows:

| | |
|---|---|
| Donor and recipient both of Group I . . . . | 0.25 o/o |
| Donor and recipient both of Group II . . . . | 16.00 o/o |
| Donor and recipient both of Group III. . . . | 1.00 o/o |
| Donor and recipient both of Group IV. . . . | 20.25 o/o |
| Total incidence where both are of same Group | 37.50 o/o |

This leaves an incidence of 62.50 o/o in which, with random mating, the possibility of isoagglutination would occur. On the assumption that the serum of only 25 o/o of human beings contains isohemolysins the chance of getting hemolysis in random matings would be reduced to 15.625 o/o.

The possibility exists that the donor's serum may hemolyze the recipient's corpuscles or that the recipient's serum may hemolyze the donor's corpuscles and in case one transfused between members of Groups II and III the serum of each may hemolyze the corpuscles of the other. Experience seems to indicate that the danger of an untoward result so far as agglutination and hemolysis are concerned is less when the donor's serum is hemolytic for the recipient's corpuscles than when the opposite condition exists. This may probably be explained as follows: The usual amount of blood transfused is 500 to 1000 cc. The total blood volume of an adult is 6000 cc. or more, therefore the donor's serum is diluted in the recipient's in the proportion of 1:6 to 1:12 assuming that both bloods are normal (as regards relative volumes of serum and red cells); the dilution being greater if the recipient is anemic, as is usually the case.

Under these conditions it is likely that any agglutinin and hemolysin which the donor's serum may contain for the corpuscles of the recipient would be diluted beyond the point at which they would act.

On the other hand the relatively small dilution of the recipient's serum by the volume of blood transfused would scarcely interfere with any action which it might have on the donor's corpuscles.

Returning to the laws of chance, the possibility that the donor's serum will contain hemolysin for the recipient's corpuscles equals the chance that the reverse relation will exist and if we assume that the former relation is not a dangerous one we may divide the figure obtained above, 15.626, by 2 and say that the percentage risk from hemolysis is 7.812. This risk is perhaps further reduced by the normal presence in the blood of an antihemolysin which acts against the normal isohemolysin. For example, if the serum of

A contains isohemolysin for the corpuscles of B, B's serum will contain an antihemolysin which protects his corpuscles from A's hemolysin. That this is not always potent to prevent hemolysis from taking place in transfusion is probably due to the fact that when the recipient's serum contains hemolysin for the donor's corpuscles, the donor's anti-hemolysin is so diluted, as explained above, by the recipient's blood that it is not present in sufficient concentration to be effective.

With random selection of donors and recipients the percentage chance that the serum of one will contain agglutinin for the corpuscles of the other is 62.50 and that the recipient's serum will contain agglutinin for the donor's corpuscles is 31.25. Moreover no normal antiagglutinin has been demonstrated in human blood for isoagglutinin. It seems highly improbable that any of the untoward results which have sometimes been observed following transfusion are due to the action of isoagglutinins.

Although the risk from hemolysins may be less than 7 per cent., in the light of our present knowledge it does not seem justifiable to disregard the blood groups in selecting donors for transfusions.

Where practicable it seems wiser to select a donor from the same isoagglutinin group as that to which the patient belongs. By doing this the possibility of isoagglutinins or isohemolysins in the serum of either for the corpuscles of the other is avoided. Such a donor may usually be easily found if the patient belongs to Group II or IV (common groups), but if the patient is a member of Group I or III it will usually be necessary to examine a larger number of donors before one of the same group is found.

In case it is not feasible to select a donor of the same group as the patient it has been suggested by Major Roger I. Lee, U. S. R., that a Group IV donor be used. Major Lee bases his suggestion on the fact that Group IV corpuscles are not agglutinated or hemolyzed by the serum of any person and that any agglutinin or hemolysin which their serum may contain for the corpuscles of the patient is so diluted by the patient's blood, as explained above, that no untoward results are liable to follow. He designates members of this group " universal donors " as they may be used for patients belonging to any or the four groups.

On the same principle members of Groups II and III may be used as donors for patients belonging to Group I.

The necessity of ruling out contageous diseases, especially syphilis, in the selection of donors and of securing robust, healthy persons need only be mentioned here.

If one were asked to formulate the conditions under which it would be justifiable to perform transfusion without previously testing the blood and selecting a donor of suitable blood group they might be stated as follows: If the danger to the life of the patient occasioned by the delay necessary for determining the blood groups is greater than the danger arising from the chance of transfusing between members of different groups, one should proceed, with the least possible delay, to transfuse without determining the group.

If one has Group II and III serum on hand the group determinations can be performed ordinarily in from ten to forty-five minutes.

In all cases where it seems probable that the necessity for transfusion may arise it is desirable to determine the blood groups of the patient and of possible donors.

In hospitals it is usually possible to have constantly on hand a number of persons who may be used as donors in case of necessity. These persons will usually be selected from patients free from communicable disease and well along in convalescence; generally those who have been lightly wounded or are recovering from the less serious operations such as for hernia. The loss of six or seven hundred cubic centimeters of blood by these patients does not materially delay convalescence and does no harm to them. The same individual will rarely be used more than once as a donor and when used more than once an interval of several weeks should, if practicable, be allowed to elapse between the first and second bleedings.

In military hospitals it does not seem advisable to use doctors, nurses, or hospital corps men as donors.

The conditions arising out of the present war have greatly increased the occasions for blood transfusion. By the simple methods now in use the operation is no longer a formidable one and it is believed that the benefits to be derived from it may be enhanced and the dangers minimized by the selection of donors from the proper blood groups.

